# *Schistosoma mansoni* Infection of Mice, Rats and Humans Elicits a Strong Antibody Response to a Limited Number of Reduction-Sensitive Epitopes on Five Major Tegumental Membrane Proteins

**DOI:** 10.1371/journal.pntd.0005306

**Published:** 2017-01-17

**Authors:** Greice Krautz-Peterson, Michelle Debatis, Jacqueline M. Tremblay, Sergio C. Oliveira, Akram A. Da’dara, Patrick J. Skelly, Charles B. Shoemaker

**Affiliations:** 1 Department of Infectious Disease and Global Health, Cummings School of Veterinary Medicine, Tufts University, North Grafton, Massachusetts, United States of America; 2 Departamento de Bioquímica e Imunologia do Instituto de Ciências Biológicas, Universidade Federal de Minas Gerais, Belo Horizonte, MG, Brazil; Queen's University Belfast, IRELAND

## Abstract

Schistosomiasis is a major disease of the developing world for which no vaccine has been successfully commercialized. While numerous *Schistosoma mansoni* worm antigens have been identified that elicit antibody responses during natural infections, little is known as to the identities of the schistosome antigens that are most prominently recognized by antibodies generated through natural infection. Non-reducing western blots probed with serum from schistosome-infected mice, rats and humans on total extracts of larval or adult schistosomes revealed that a small number of antigen bands predominate in all cases. Recognition of each of these major bands was lost when the blots were run under reducing condition. We expressed a rationally selected group of schistosome tegumental membrane antigens in insect host cells, and used the membrane extracts of these cells to unambiguously identify the major antigens recognized by *S*. *mansoni* infected mouse, rat and human serum. These results revealed that a limited number of dominant, reduction-sensitive conformational epitopes on five major tegumental surface membrane proteins: SmTsp2, Sm23, Sm29, SmLy6B and SmLy6F, are primary targets of mouse, rat and human *S*. *mansoni* infection sera antibodies. We conclude that, *Schistosoma mansoni* infection of both permissive (mouse) and non-permissive (rat) rodent models, as well as humans, elicit a dominant antibody response recognizing a limited number of conformational epitopes on the same five tegumental membrane proteins. Thus it appears that neither infecting schistosomula nor mature adult schistosomes are substantively impacted by the robust circulating anti-tegumental antibody response they elicit to these antigens. Importantly, our data suggest a need to re-evaluate host immune responses to many schistosome antigens and has important implications regarding schistosome immune evasion mechanisms and schistosomiasis vaccine development.

## Author Summary

Schistosomiasis is caused by blood flukes residing in the veins of infected individuals and afflicts millions of people in the developing world. The schistosome worms can remain healthy in the bloodstream for more than 10 years, implying an extraordinary ability to evade host immune damage. Scientists are seeking to understand immune evasion so as to find weaknesses in defenses that can be exploited in the development of effective vaccines. Here we investigate the normal antibody response to schistosomes during infection of mice, rats and humans, and show for the first time that this response is highly skewed to the recognition of a small number of proteins present at the worm surface. Surprisingly, these abundant antibodies recognize their targets only when the proteins retain their native conformations, stabilized by the presence of intramolecular disulfide bridges. Because of this conformational-dependence, these antibodies have remained undetected in prior studies in which antibody binding assays were routinely performed in a reducing environment that destroys disulfide bridges. The routine presence of these antibodies within the serum of schistosome infected patients and animals raises new and interesting questions as to their possible role in immune evasion, and has significant implications for schistosomiasis vaccine development.

## Introduction

Schistosomiasis is a disease affecting more than 200 million individuals living mostly in underdeveloped tropical and subtropical regions (http://www.who.int/mediacentre/factsheets/fs115/en/). The disease is caused by infection with schistosome blood flukes which can survive, if untreated, for decades inside the vascular system of immune competent permissive hosts. Illness is primarily a consequence of immunopathology from schistosome eggs trapped in tissues (reviewed by [1]). Despite the long term presence of adult worms in their vascular system, permissive hosts, by definition, do not typically develop immune responses directed at juvenile or adult worms [2] that are capable of preventing new infections or eliminating all adult worms. This is not to imply that anti-worm immune responses are completely ineffective, as a measure of protective immunity following multiple rounds of reinfection has been documented [3, 4].

The humoral response to schistosome infection has been extensively characterized in efforts to identify antigens for use in the diagnosis of infection or that might elicit a response that protects vaccinated individuals from infection. To seek protective vaccine candidates, researchers have sought to identify antigens that are recognized more intensely by serum antibodies from animal models with demonstrated resistance to infection. Examples include comparisons in schistosome antigen recognition by: 1) non-permissive rats vs permissive mice models [5, 6]; 2) mice +/- vaccination by irradiated cercariae [7], and; 3) humans displaying susceptibility vs putative resistance to infection [8, 9]. Through these efforts, dozens of schistosome antigens have been found to be recognized by antibodies in serum from infected humans or rodents (reviewed by [10–12]). While these efforts have identified antigens recognized by various infection sera, it remains unclear as to which antigens are most abundantly targeted by antibodies in these sera. This information could aid in understanding the nature of the normal humoral immune response to schistosome infection and provide important guidance to efforts seeking improved diagnostic tools and new vaccines.

In an effort to identify which proteins on larval and adult schistosomes are most abundantly recognized by antibodies in serum of infected rodents and humans, we began by probing western blots with these sera on total schistosome antigen extracts. To better preserve antigen conformation, we did not destroy protein disulfide bridges by chemical reduction prior to resolution on gels. Highly diluted sera from schistosome infected rats, mice and humans (1:2000) all, surprisingly, produced similar banding patterns on blots of total extracts of schistosomula and adults, revealing a remarkably small number of antigen bands that predominate. When the antigen samples were reduced, these major bands were no longer observed by the same diluted sera.

Based on a variety of inferences, explained below, we selected a set of candidate schistosome antigens which were then expressed within insect cells. Here we use extracts of these cells to identify two tetraspanins: SmTsp2 and Sm23 and three Ly6 family proteins: Sm29, SmLy6B, and SmLy6F, as the major schistosome antigens recognized by the antibodies in dilute serum of infected mice, rats and humans. All of these proteins are reported to be present within the host-interactive surface tegument of schistosomes, and two of the antigens, SmTsp2 and Sm29, are currently strong candidates as vaccine immunogens to prevent schistosomiasis [8, 13, 14]. We show that the infection serum antibodies recognizing these two antigens can be largely blocked by two previously reported monoclonal single-chain Fv domain antibodies (scFvs Teg1 and Teg4 [15]), which suggests that the serum primarily recognizes limited epitopes on these antigens. These findings have significant implications regarding both the mechanisms by which schistosomes evade immune damage and the development of schistosomiasis vaccines, which are discussed.

## Results

### Western blots of total larval and adult *S*. *mansoni* extracts probed by sera from recently infected mice, rats or humans

**Fig 1:**
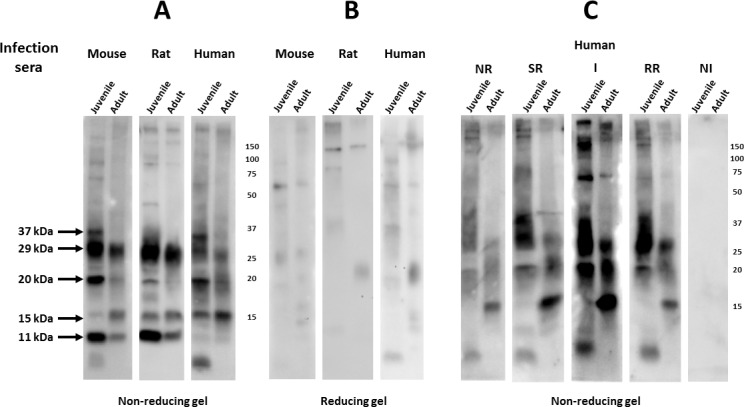
Serum from *S*. *mansoni* infected mice, rats and humans recognize a limited number of reduction-sensitive epitopes on western blots of total worm extracts. Total extracts from juvenile (5 day cultured schistosomula) or adult mammalian-stage *Schistosoma mansoni* worms were resolved by SDS-PAGE under reducing (0.1% βME) or non-reducing conditions and transferred to filters. Filters were probed with serum from mice, rats or humans (1:2000) that were infected by *S*. *mansoni*. Detection employed the appropriate HRP/anti-mouse, -rat or -human IgG reagent. A. Filters from a non-reducing gel probed by pooled sera from ten chronically infected mice; pooled sera from five, twice-infected Fischer rats, or; pooled serum from 8 human patients identified as having schistosomiasis. B. Filters from a reducing gel probed with the same sera employed in A. C. Filters from a non-reducing gel probed with five different pools of human sera (NR, SR, I, RR, NI), each derived from eight patients displaying common apparent susceptibility or resistance to schistosome re-infection (for details on these sera, see Pinheiro et al, 2014 [13]). Numbers represent molecular mass markers (kDa).

Total antigen extractions were performed on larval and adult schistosomes by boiling the freshly frozen parasites in an SDS gel loading buffer under non-reducing conditions. Serum was typically diluted to 1:2000 so as to preferentially reveal the antigens recognized by the more prevalent anti-schistosome antibodies. Fig 1A shows that similar patterns are recognized on Western blots prepared from either juvenile (5 day cultured schistosomula) or adult worm extracts when probed with pooled sera prepared from *S*. *mansoni*-infected mice, rats and humans (‘infection sera’). All three infection sera recognize antigens of the same apparent MW, though with differences in intensity, on both juvenile and adult worm extracts. The predominant staining species were estimated to be about 11, 15, 20, 29 and 37 kDa (arrows, Fig 1A) by comparison to molecular weight standards.

Surprisingly, recognition of these major bands is largely lost from the infection sera when the same schistosome extracts are resolved on western blots under reducing conditions at 0.1% β-mercaptoethanol (βME) (Fig 1B). A similar dramatic reduction in western blot signals produced by mouse, rat or human infection sera was also observed when the (non-reducing gel) filters were pre-washed with 0.1% βME prior to serum incubation (not shown). These results demonstrate that the major epitopes on the dominant schistosome antigens present in both larval and adult schistosome extracts are reduction-sensitive, and thus likely to be conformational epitopes dependent on disulfide cross-linkages.

Studies were also performed to test the consistency of the infection sera western blot data when using independent pools of serum, and to compare sera from human patients having different infection histories. First, during the course of our studies we generated a second pool of mouse infection sera and found antigen recognition within juvenile and adult schistosome extracts to be virtually indistinguishable and both sera were used interchangeably (not shown). Next we compared four different serum pools from patients with different schistosome infection histories and different apparent susceptibilities to infection (see [13]). Two of the pools were obtained from patients that had active schistosome infections (I, infected; SR, susceptible to reinfection) and two pools from patients that had frequent exposures to cercariae and yet were currently uninfected (RR, resistant to reinfection; NR, natural resistant). Unlike the I and NR groups, the SR and RR patients had been recently treated with praziquantel. A fifth pool of serum (NI, noninfected) came from people having no history of schistosome exposure. Each group contained both male and female donors. As shown in Fig 1C, while the pools produced variable signal intensities, all pools produced similar banding patterns. Non-infected patient sera completely lack any signal, demonstrating the absence of background under the conditions used. Overall, these results suggest that the schistosome antigen recognition patterns reported here with infection sera, when resolved on gels under non-reducing conditions, are almost certainly representative of infection sera from other rodent and human individuals, even those having differences in their infection histories. Thus, we conclude that the dominant recognition of these five schistosome antigen bands appear to be generally consistent across various host genetic backgrounds and parasite exposure histories.

**Fig 2:**
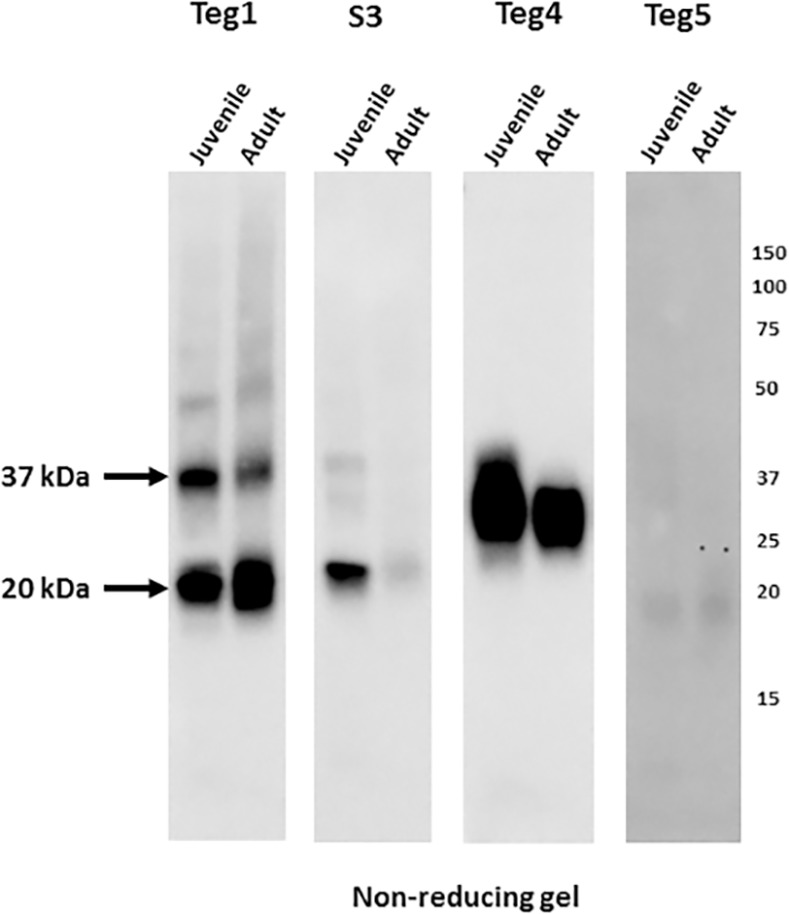
Anti-tegumental rat scFvs recognize distinct juvenile and adult *S*. *mansoni* antigens when resolved on non-reducing gels. Total extracts from juvenile or adult mammalian-stage *Schistosoma mansoni* worms were resolved by SDS-PAGE under non-reducing conditions and transferred to filters. Filters were probed by one of four different anti-tegumental scFvs (Teg1, S3. Teg4 and Teg5, all at 1 μg/ml) previously obtained from *S*. *mansoni* infected rats. Detection was by HRP/anti-E-tag recognition of a peptide E-tag expressed with each scFv. Numbers indicate the positions of migration of molecular mass markers (kDa).

We previously reported [15] the isolation of four recombinant single-chain Fv proteins (scFvs) recognizing surface antigens on live schistosomes that we obtained from the B cells of schistosome infected rats, three of which recognize living schistosomula (Table 1). Two of the scFvs were shown to recognize known tegumental antigens; Teg1 recognizes the tetraspanin SmTsp2 [16], Teg4 recognizes Sm29 [17] (also called SmLy6D [18]). A third scFv (Teg5) was shown to weakly recognize living schistosomes, and an scFv called S3 had been obtained earlier from the same source [19] which recognizes the schistosome tetraspanin antigen, Sm23. When schistosome extracts resolved by non-reducing gels were probed by western blotting with these scFvs, several produced strong signals (Fig 2). These signals were dramatically diminished or undetectable when the extracts were resolved on reducing gels (not shown). Of interest, each of the scFvs recognized antigens having the same mobility as antigens recognized by the various (mouse, rat, human) infection sera depicted in Fig 1. Teg1 and S3 recognized their tetraspanin targets (Tsp2 and Sm23) as 20 and 37 kDa species (Fig 2, arrows). The presence of the larger species suggests that the ~20 kDa monomers become partially cross-linked into ~37 kDa dimeric forms under the non-reducing conditions. Teg4 strongly recognized the 29 kDa Sm29 while Teg5 recognized a minor antigen of about 20 kDa.

**Table 1:** Summary of anti-tegumental rat scFvs.

scFv name	*S*. *mansoni* antigen	Binding to surface of live schistosomula
S3	Sm23^[^^19^^]^	No^b^
Teg1	SmTsp2^[^^15^^]^	Yes^[^^15^^]^
Teg4	Sm29^[^^15^^]^	Yes^[^^15^^]^
Teg5	SmLy6C^a^	Yes^[^^15^^]^

^a^Identified in this report (Fig 4).

^b^No staining observed with this scFv.

### Identification of the major antigens recognized by schistosome infection sera–candidate antigen selection

To identify the reduction-sensitive schistosome antigens from larval and adult worm extracts that are recognized by dilute schistosome infection sera on Western blots, we sought to produce conformationally-native recombinant versions of a set of selected candidate antigens and test their ability to specifically block infection sera antibodies recognizing one or more of the major bands. Three obvious candidate antigens were SmTsp2, Sm23 and Sm29 based on the fact that our prior studies had identified them as targets of scFvs that were obtained from B cells of schistosome-infected rats [15], and the fact that the gel mobility of their target antigens matched those of the three larger antigens (20, 29, 37 kDa) recognized by infection sera (Figs 1 and 2).

The two other major bands recognized by schistosome infection sera had apparent MWs of 11 or 15 kDa. This small size is similar to that of most members of the SmLy6 family of membrane proteins [18, 20]. The Ly6 proteins are GPI-anchored proteins, some of which are known to be present in the schistosome tegument [20, 21], and all are expected to have conserved, reduction-sensitive, intra-chain disulfide linkages within their extracellular domains [22]. Several of the smaller SmLy6 proteins are also known to be well-expressed by mammalian stage schistosomes [20]. In fact, Sm29 is also a Ly6 family protein (SmLy6D) containing two linked Ly6 domains [18]. Based on their small size, sensitivity to reduction and available expression data, we selected four additional members of the SmLy6 family: SmLy6A, SmLy6B, SmLy6C and SmLy6F (nomenclature from [18]) as strong candidates to be major antigens recognized by schistosome infection sera. These four SmLy6 proteins were also called SmCD59.1, SmCD59.2, SmCD59.3 and SmCD59.4, respectively, in another recent report on putative schistosome membrane antigens [20].

### Recognition of selected schistosome membrane proteins by infection sera

Early efforts to produce recombinant schistosome SmTsp2 and Sm29 proteins were performed in *E*. *coli* host cells transformed with expression vectors containing the extracellular regions of these proteins fused to *E*. *coli* thioredoxin (Trx) to facilitate protein folding. Western blots with these purified recombinant proteins were poorly recognized by rat infection sera or the scFvs, probably because the reduction-sensitive epitopes were not reproduced well when expressed in *E*. *coli* cytosol. We thus began employing the baculovirus expression system within insect cell hosts in an effort to generate recombinant membrane protein antigens that we expected would better retain the native conformations of these proteins on schistosomes.

The seven rationally selected schistosome membrane protein candidates (above) were each expressed in recombinant insect cells as described in Materials and Methods. The SmLy6 proteins (SmLy6A, SmLyB, SmLy6C and SmLy6F) were expressed with an insect secretory leader and each contained a FLAG-tag epitope (DYKDDDDK) that remained at their amino ends following signal processing. The tetraspanin proteins, SmTsp2 and Sm23 were expressed using their complete *S*. *mansoni* coding DNA. Sm29 was also expressed with its natural signal peptide and using full-size coding DNA. Recombinant insect cells expressing the schistosome antigens were harvested and the membrane proteins solubilized in a lysis buffer containing 1% Triton X-100 and no reducing agents.

**Fig 3:**
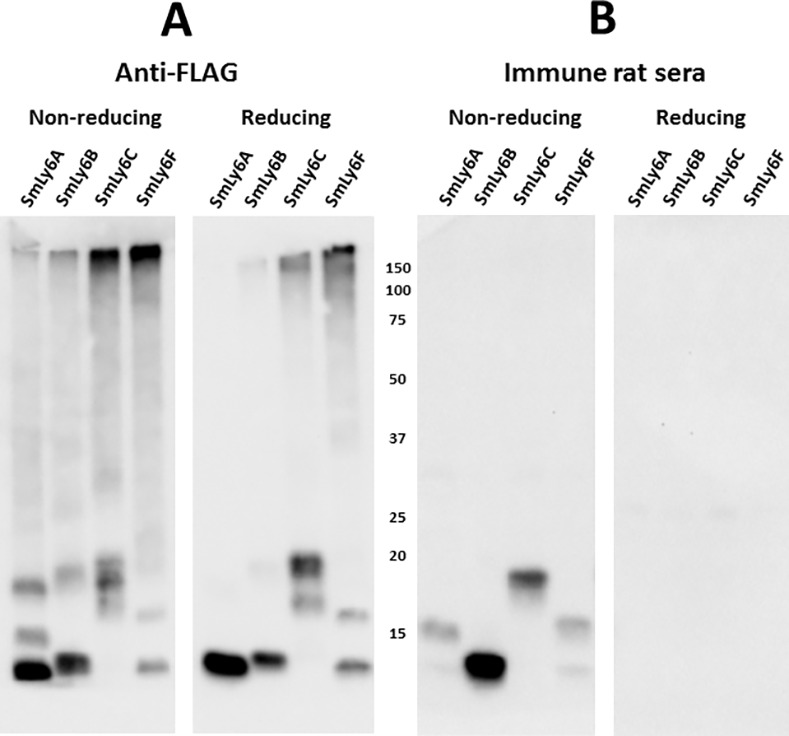
*S*. *mansoni* infected rat sera recognize reduction-sensitive epitopes in recombinant insect cell extracts containing SmLy6 proteins. Insect cells engineered to express SmLy6A, SmLy6B, SmLy6C and SmLy6F were extracted in a mild detergent buffer and resolved by SDS-PAGE under non-reducing or reducing conditions, as indicated. Filters with insect cell extracts containing recombinant SmLy6A, B, C or F were probed by anti-FLAG (**A**) or rat infection sera (1:2000) (**B**). Detection employed HRP/anti-FLAG tag or HRP/anti-rat IgG. Numbers indicate the positions of migration of molecular mass markers (kDa).

Western blots were prepared from insect cell extracts containing the four small recombinant SmLy6 proteins (SmLy6A, B, C, F) and then probed with anti-FLAG antibody (Ab). As shown in Fig 3A, under non-reducing conditions the insect cell extracts expressing SmLy6A, B and F contained FLAG-tagged proteins in the expected size range (~12 kDa). For each of the extracts, one or two larger FLAG protein species were also present in the 15–20 kDa size range. Unexpectedly, insect SmLy6C extracts did not contain a FLAG protein of the expected size, but instead contained several bands clustered about 19 kDa. Higher molecular weight forms were visible with all four SmLy6 proteins. When the SmLy6 extracts were resolved on gels under reducing conditions, the lower MW bands remained while most of the 15–20 kDa bands and higher MW forms were no longer visible, suggesting that the higher MW forms are multimers stabilized by disulfide linkages.

The same four recombinant SmLy6 insect cell extracts were also probed with pooled *S*. *mansoni* rat infection sera (Fig 3B). All four extracts contained a protein species recognized by the rat sera that was a subset of bands recognized by anti-FLAG, indicating that only some of the recombinant protein had folded to a conformation recognized by the infection sera. Surprisingly, for SmLy6A, C and F, the bands primarily recognized by immune rat sera were the MW species ~15–20 kDa. These bands are larger than expected and may be glycosylated. Only the SmLy6B extract contained a band of ~11 kDa, the expected size of these SmLy6 proteins based on coding sequence, which was clearly recognized by the rat infection sera. As when probing the schistosome extracts (Fig 1), rat infection sera recognition of the four recombinant SmLy6 proteins was lost when the gel was run under reducing conditions (Fig 3B, right panel).

**Fig 4:**
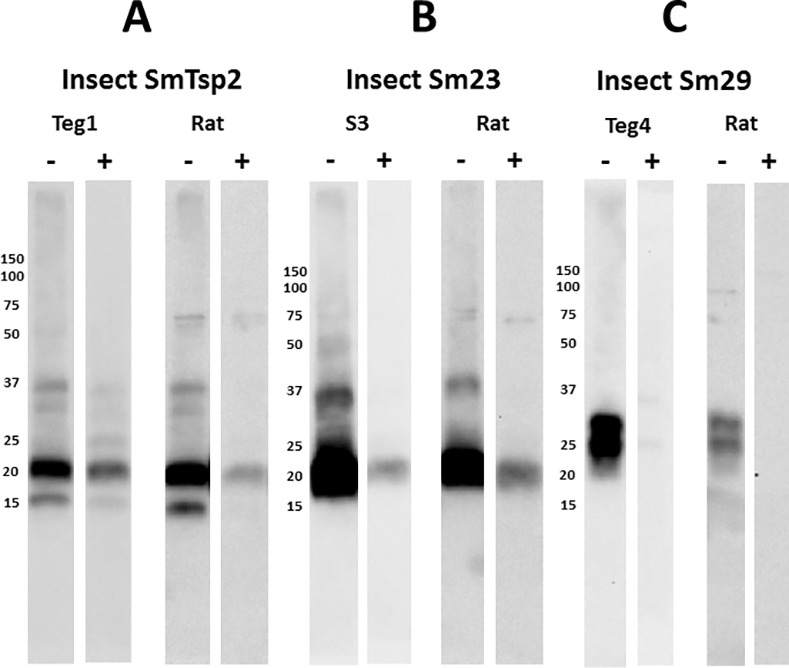
*S*. *mansoni* infected rat sera recognize reduction-sensitive epitopes in recombinant insect cell extracts containing SmTsp2, Sm23 or Sm29. Insect cells engineered to express selected SmTsp2, Sm23 or Sm29 were extracted in a mild detergent buffer and resolved by SDS-PAGE under non-reducing (-) or reducing (+) conditions. **A**. Filters containing insect cell extracts containing SmTsp2 were probed with Teg1 scFv or rat infection sera (1:2000). Detection employed HRP/anti-E-tag for Teg1 scFv, or HRP/anti-rat IgG. **B**. Filters containing insect cell extracts containing Sm23 were probed with S3 scFv or rat infection sera (1:2000). Detection employed HRP/anti-E-tag for S3 scFv, or HRP/anti-rat IgG. **C**. Filters containing insect cell extracts containing Sm29 were probed with Teg4 scFv or rat infection sera (1:2000). Detection employed HRP/anti-E-tag for Teg4 scFv, or HRP/anti-rat IgG. Numbers indicate the positions of migration of molecular mass markers (kDa).

Insect extracts expressing SmTsp2, Sm23 and Sm29, which lack a FLAG tag, were resolved on non-reducing or reducing gels and probed with the appropriate scFvs (Teg1, S3, Teg4 respectively) or with rat infection sera (Fig 4). As expected, the three scFvs recognized their insect cell expressed target antigens. Rat infection sera produced a virtually identical recognition pattern as the scFvs on replicate non-reducing blots, indicating, as has been previously reported, that schistosome infection sera contain antibodies recognizing SmTsp2, Sm23 and Sm29 [8, 9, 23]. Most or all recognition of these proteins by the scFvs or the infection serum was lost when these recombinant insect extracts were resolved under reducing conditions.

**Fig 5:**
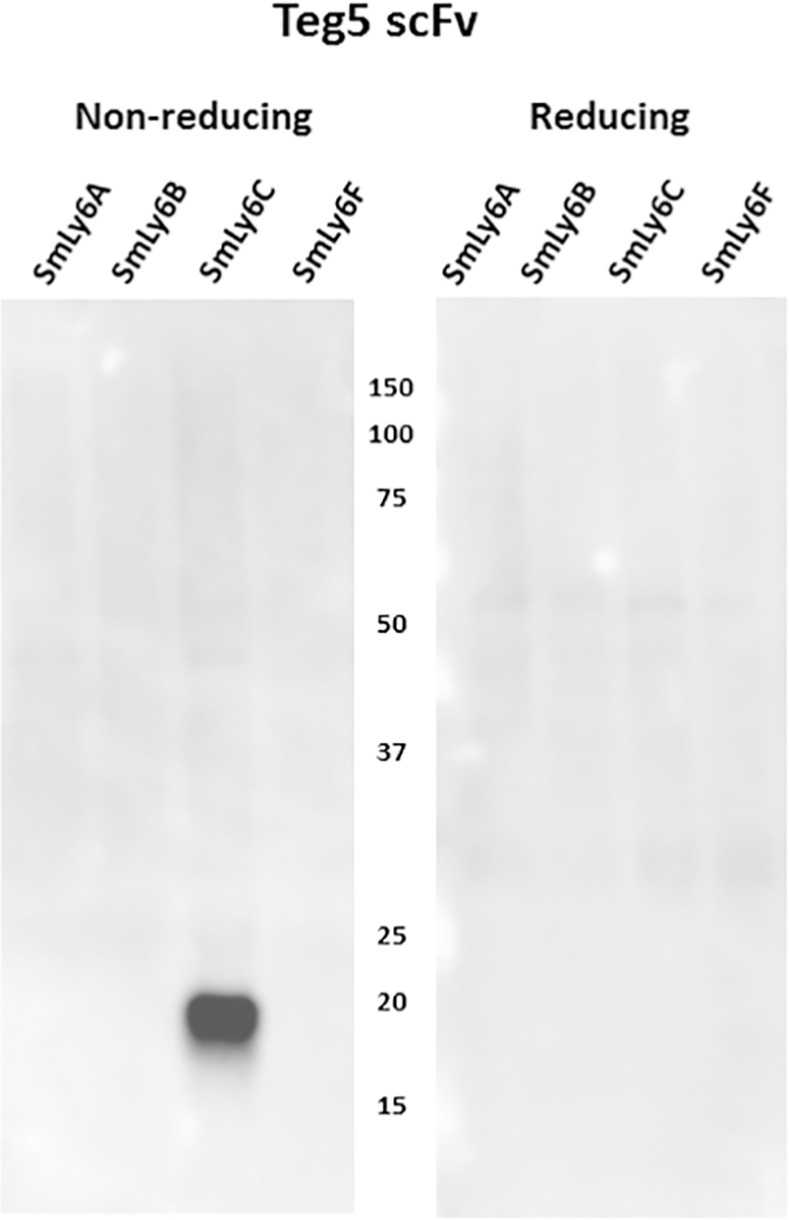
Anti-*S*. *mansoni* tegument scFv, Teg5, recognizes reduction-sensitive epitopes on SmLy6C. Insect cells engineered to express recombinant SmLy6A, B, C or F were extracted in a mild detergent buffer and resolved by SDS-PAGE under non-reducing or reducing conditions and transferred to filters. Filters were probed by Teg5 scFv and recognition detected with HRP/anti-E-tag. Numbers indicate the positions of migration of molecular mass markers (kDa).

We also tested Teg5 scFv for recognition of the insect cell extracts containing the various *S*. *mansoni* membrane proteins. As shown in Fig 5, Teg5 clearly recognizes a reduction-sensitive epitope on SmLy6C. Since Teg5 was shown to recognize living worms [15], these results shows that SmLy6C can be expressed on the outside surface of larval and adult schistosomes. Thus Teg5, like Teg1 and Teg4, recognize conformational epitopes on schistosome tegumental antigen targets, consistent with the finding that rat infection sera predominantly recognize reduction-sensitive epitopes on schistosome antigens.

**Fig 6:**
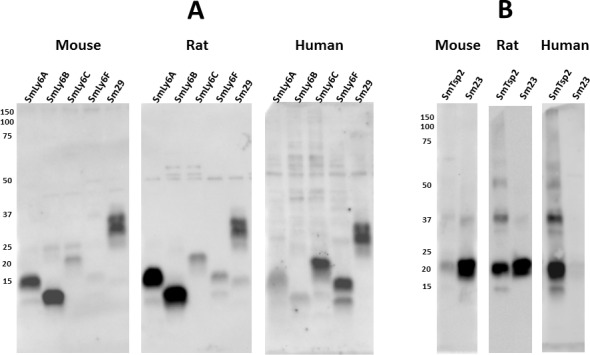
*S*. *mansoni* infected mouse, rat and human sera recognition of selected *S*. *mansoni* antigens expressed as recombinant proteins by insect cells. Insect cells engineered to express selected *S*. *mansoni* membrane antigens were extracted in a mild detergent buffer and resolved by SDS-PAGE under non-reducing conditions. **A.** Replicate filters containing SmLy6A, SmLy6B, SmLy6C, SmLy6F and Sm29 were probed by mouse, rat or human infection sera (1:2000). **B**. Replicate filters containing SmTsp2 and Sm23 were probed by mouse, rat or human infection sera. Detection employed the appropriate HRP/anti-mouse, -rat or -human IgG reagent. Numbers indicate the positions of migration of molecular mass markers (kDa).

Each of the insect extracts expressing the selected schistosome membrane proteins were characterized by non-reducing Western blots for their recognition by serum from infected rats, mice and humans. Blots with the five SmLy6 proteins are shown in Fig 6A and reveal that rat, mouse and human infection sera recognize each of the proteins, though with variable intensity. In general, mice and rats recognize SmLy6A and SmLy6B more intensely than SmLy6C and SmLy6F, while the reverse was true with the human infection serum pool. All infection sera recognized Sm29 to a similar extent. Results with the two tetraspanin proteins, SmTsp2 and Sm23, were more variable (Fig 6B). When quantified by imaging, the rat infection serum pools recognized both proteins about equally, while the mouse pool recognized Sm23 with >10x greater intensity than SmTsp2 and the human serum pool recognized SmTsp2 with >10x greater intensity than Sm23.

### Identification of major *S*. *mansoni* antigens by infection sera pre-incubation with recombinant proteins

**Fig 7:**
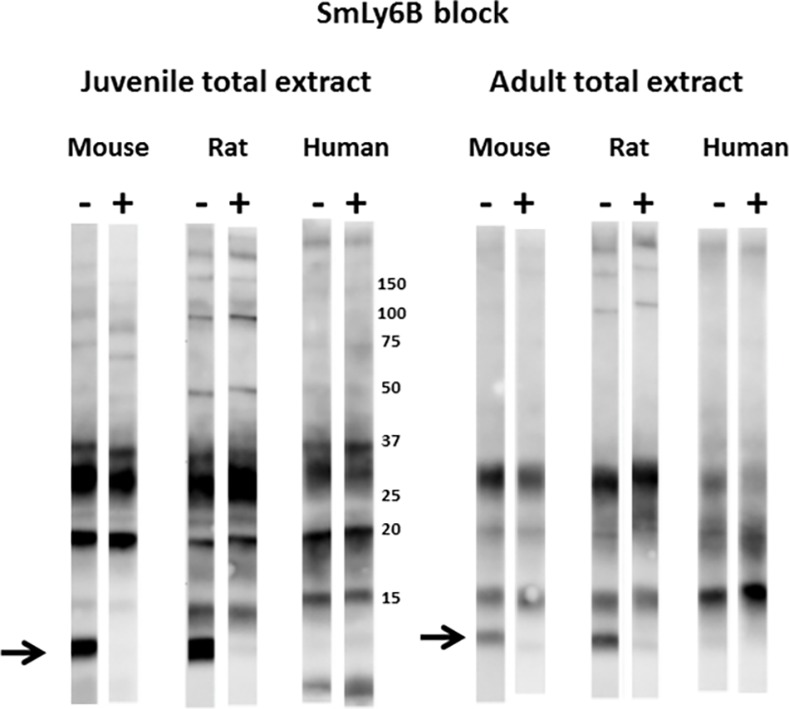
Insect extracts containing recombinant SmLy6B block recognition of a major 11 kDa reduction-sensitive *S*. *mansoni* antigen on western blots by mouse and rat infection sera. Total extracts from juvenile or adult mammalian-stage *Schistosoma mansoni* worms were resolved by SDS-PAGE under non-reducing conditions and transferred to filters. Pooled mouse, rat or human infection sera (1:2000) were pre-incubated for 1 hr with control lysate buffer (-) or lysates of insect cells expressing SmLy6B (+) prior to overnight incubation with the filter strips. Detection employed the appropriate HRP/anti-mouse, -rat or -human IgG reagent. The arrow indicates the position of migration of SmLy6B. Numbers indicate the positions of migration of molecular mass markers (kDa).

**Fig 8:**
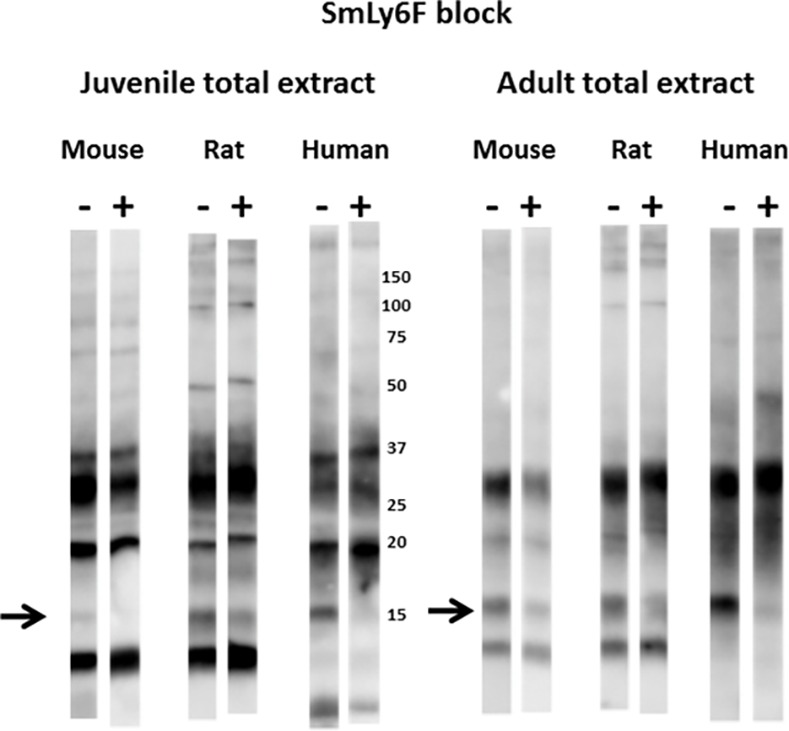
Insect extracts containing recombinant SmLy6F block recognition of a major 15 kDa reduction-sensitive *S*. *mansoni* antigen on western blots by mouse, rat and human infection sera. Total extracts from juvenile or adult mammalian-stage *Schistosoma mansoni* worms were resolved by SDS-PAGE under non-reducing conditions and transferred to filters. Pooled mouse, rat or human infection sera (1:2000) were pre-incubated for 1 hr with control lysate buffer (-) or lysates of insect cells expressing SmLy6F (+) prior to overnight incubation with the filter strips. Detection employed the appropriate HRP/anti-mouse, -rat or -human IgG reagent. The arrow indicates the position of migration of SmLy6F. Numbers indicate the positions of migration of molecular mass markers (kDa).

**Fig 9:**
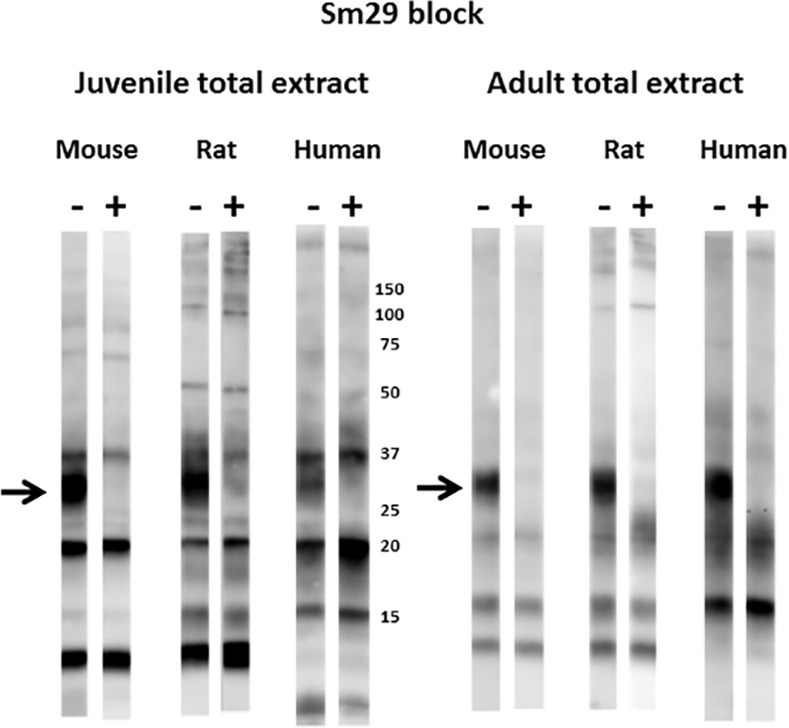
Insect extracts containing recombinant Sm29 block recognition of a major 29 kDa reduction-sensitive *S*. *mansoni* antigen on western blots by mouse, rat and human infection sera. Total extracts from juvenile or adult mammalian-stage *Schistosoma mansoni* worms were resolved by SDS-PAGE under non-reducing conditions and transferred to filters. Pooled mouse, rat or human infection sera (1:2000) were pre-incubated for 1 hr with control lysate buffer (-) or lysates of insect cells expressing Sm29 (+) prior to overnight incubation with the filter strips. Detection employed the appropriate HRP/anti-mouse, -rat or -human IgG reagent. The arrow indicates the position of migration of Sm29. Numbers indicate the positions of migration of molecular mass markers (kDa).

**Fig 10:**
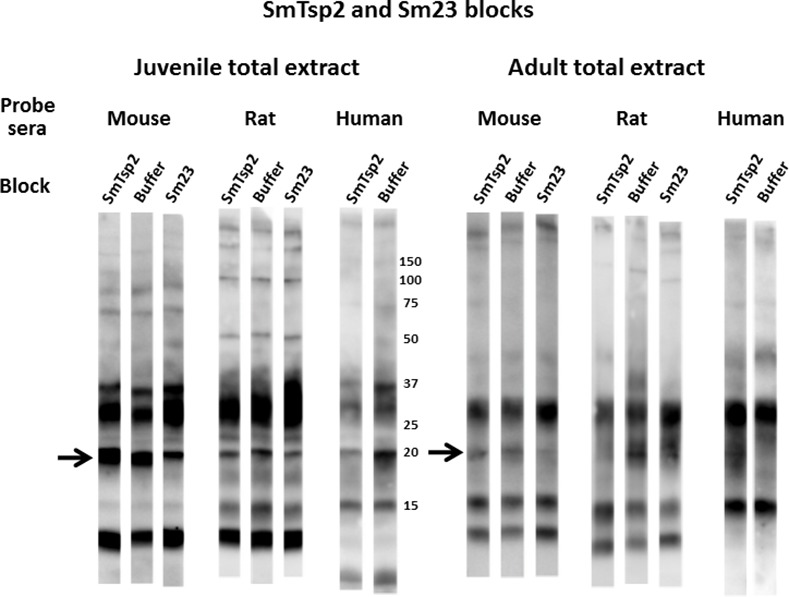
Insect extracts containing recombinant tetraspanins, SmTsp2 and Sm23 block recognition of a major 20 kDa reduction-sensitive *S*. *mansoni* antigen on western blots by mouse, rat and human infection sera. Total extracts from juvenile or adult mammalian-stage *Schistosoma mansoni* worms were resolved by SDS-PAGE under non-reducing conditions and transferred to filters. Pooled mouse, rat or human infection sera (1:2000) were pre-incubated for 1 hr with control lysate buffer or lysates of insect cells expressing SmTsp2 or Sm23 (as indicated) prior to overnight incubation with the filter strips. Detection employed the appropriate HRP/anti-mouse, -rat or -human IgG reagent. The arrow indicates the position of migration of SmTsp2 and Sm23. Numbers indicate the positions of migration of molecular mass markers (kDa).

Mild detergent extracts of insect cells expressing a single recombinant schistosome antigen were next tested for their ability to block recognition of specific bands produced by various infection sera on western blots of SDS-PAGE resolved juvenile and adult schistosome extracts (Figs 7–10). The insect cell extracts from all of the seven rationally selected schistosome membrane antigens were separately incubated with mouse, rat or human infection sera prior to and during the incubation of the sera with western blots of total extracts of juvenile and adult schistosomes. All the results shown were repeated at least three times, and compared two different pools of mouse infection sera and four different pools of human infection sera over the course of these studies, and yet produced consistent results. In some cases, we were unable to completely block recognition of a specific band through pre-incubation with an insect cell extract, and it is possible in these cases that the insect cell recombinant protein lacks a conformer of the target antigen which is present in schistosome extracts, or that a second, unidentified minor antigen having the same gel mobility co-migrates with the major antigen species.

Figs 7 and 8 show that pre-incubation of rodent or human infection serum pools with insect cell extracts containing recombinant SmLy6B or SmLy6F specifically absorbed antibodies that recognize the 11 kDa or 15 kDa bands, respectively. Insect SmLy6B blocked the strong recognition of an 11 kDa band by infection sera from both of the rodent sera (Fig 7). A band at 11 kDa was also weakly recognized by human infection sera and this band was reproducibly blocked by SmLy6B in similar blots, though it is not clear in the example shown. Insect extracts containing SmLy6F consistently blocked recognition of the 15 kDa band that is well recognized by rodent and human infection sera (Fig 8). This antigen was not expressed well by insect cells and the blocking studies were less robust, yet we consistently observed reduction in the 15 kDa signals produced by all the infection sera. These results show that 11 kDa and 15 kDa schistosome antigens prominently recognized by infection sera on western blots of schistosome total extracts are SmLy6B and SmLy6F.

We were repeatedly unable to observe a reduction in the recognition of any of the antigens when the three infection sera pools were pre-incubated with extracts containing SmLy6A or SmLy6C (not shown). Negative results in these studies though are inconclusive due to uncertainty as to whether sufficient insect antigen, in its properly folded state, is present in the insect cell extracts to successfully block the infection serum antibodies. Nevertheless, since the rat infection sera recognize recombinant SmLy6A and SmLy6C (Fig 3B), these results suggest that these antigens may not be sufficiently well expressed by larval or adult schistosomes to produce major bands on Western blots of total worm extracts.

Serum blocking studies with Sm29, shown in Fig 9, were particularly dramatic and robust. The major 29 kDa antigen, which is present in both schistosomula and adult schistosomes and recognized by mouse, rat and human infection sera, is almost quantitatively quenched when the sera are pre-incubated with recombinant Sm29 extracts. Antibody recognition of Sm29 has proven to be extremely sensitive to reduction and other denaturing conditions (not shown), yet when care is taken in the preparation of schistosome extracts, Sm29 has consistently been observed as one of the two or three most dominant antigens recognized by infection sera from mice, rats and humans.

Similar blocking studies were also performed with insect extracts expressing SmTsp2 or Sm23 (Fig 10). The major 20 kDa band recognized by all three infection sera was variably blocked by the presence of these extracts, likely because of the large variation in the titers of anti-SmTsp2 and anti-Sm23 antibodies in mouse vs human infection sera (Fig 6B). While both extracts seemed to consistently diminish the 20 kDa signal produced by rat infection sera, only the Sm23 extracts had this effect on mouse infection sera. The human infection serum recognition of the 20 kDa band was largely and consistently blocked by incubation with SmTsp2 extracts. Sm23 extracts were not tested due to the negligible titer of Sm23 antibodies in human sera (Fig 6B). We conclude that the major 20 kDa species recognized by the three infection sera is likely a mixture of both SmTsp2 and Sm23, and perhaps other proteins of this size such as other known tetraspanins [24].

### Schistosome infection sera primarily recognize a single conformational epitope on SmTsp2 and Sm29

**Fig 11:**
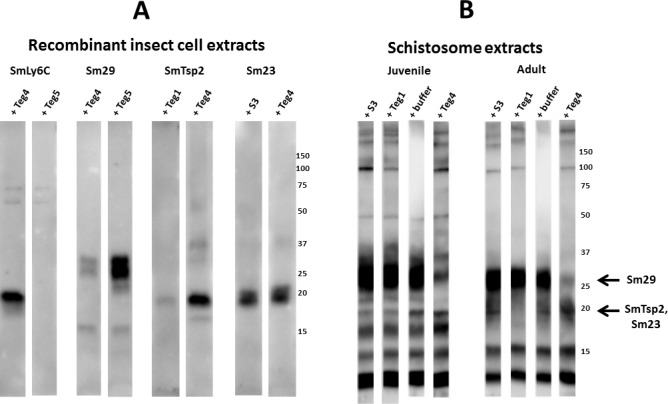
*S*. *mansoni* infected rat sera recognition of SmLy6C, SmTsp2 and Sm29 can be substantially suppressed by a single monoclonal scFv antibody. Detergent extracts of recombinant insect cells (A), or total extracts from juvenile or adult mammalian-stage *Schistosoma mansoni* worms (B) were resolved by SDS-PAGE under non-reducing conditions and transferred to filters. Replicate filter strips containing the indicated extracts were pre-incubated with the indicated scFv (Teg1, Teg4, Teg5 or S3, all at 10 μg/ml) or with buffer alone for 1 hr with *S*. *mansoni* rat infection sera (1:2000) prior to overnight incubation with the filter strips. Detection employed HRP/anti-rat IgG. Arrow indicates the position of migration of Sm29 and SmTsp2.Sm23. Numbers indicate the positions of migration of molecular mass markers (kDa).

We next tested whether our four anti-schistosome scFvs (Teg1, S3, Teg4 and Teg5) could effectively compete with antibodies in infection sera for binding to their antigen targets. In Fig 11A, we individually tested our monoclonal scFvs for their ability to interfere with rat infection sera binding to their targets expressed by insect cells. Surprisingly, Teg5, Teg4, and Teg1, were able to almost completely block rat infection sera recognition of SmLy6C, SmTsp2 and Sm29 respectively, while pre-incubation of the filters with a different scFv had no effect. Only S3 scFv was unable to appreciably diminish recognition of its target, Sm23, by the rat sera.

We next tested the ability of the scFvs to block rat infection sera recognition of specific bands within the juvenile and adult schistosome total extract (Fig 11B). The Teg1 and S3 scFvs both appeared to diminish recognition of the 20 kDa species in juvenile schistosomula, while only Teg1 had this effect in adult extracts. This is consistent with the results in Fig 2 showing that Sm23 is much more abundant than SmTsp2 in schistosomula than in adults. The results suggest that the Teg1 monoclonal scFv blocks most or all rat infection sera recognition of SmTsp2. This blocking effect was even more obvious with Teg4 pre-incubation which produced a major reduction in the rat sera recognition of the 29 kDa band identified as Sm29. Together these results suggest that the Teg1 and Teg4 scFvs bind epitopes that represent the dominant epitope for rat infection sera binding to either SmTsp2 or Sm29.

**Fig 12:**
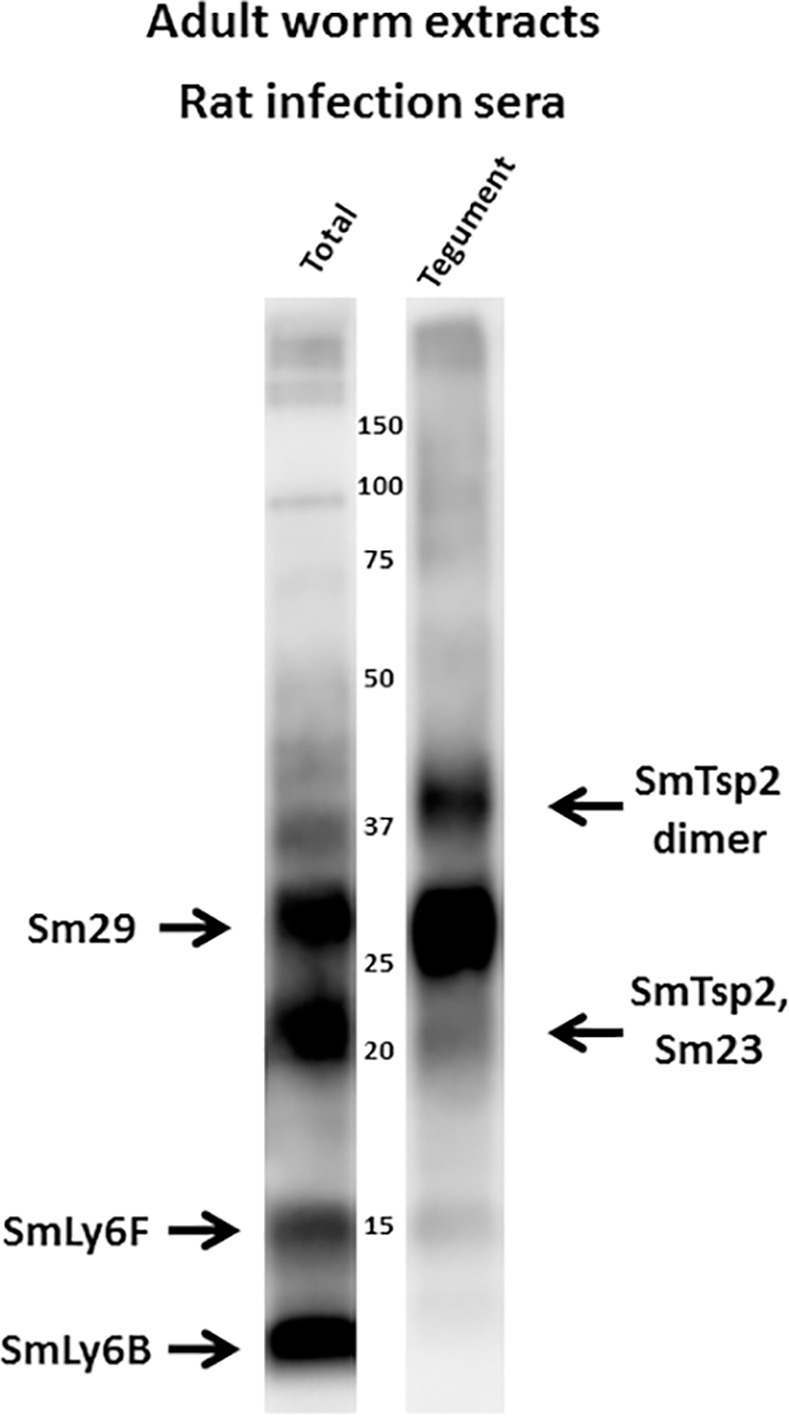
*S*. *mansoni* infected rat sera recognition of total vs tegumental extracts of *S*. *mansoni* worms. Total extracts or tegumental extracts of adult mammalian-stage *S*. *mansoni* worms were resolved by SDS-PAGE under non-reducing conditions and transferred to filters. Filters were probed with rat infection sera. Major bands are identified (arrows) based on studies reported in this manuscript. Numbers indicate the positions of migration of molecular mass markers (kDa).

Fig 12 shows a Western blot comparing rat infection sera recognition of adult worm total extracts with an adult worm extract that is highly enriched in tegumental proteins [15, 25]. The figure summarizes the identities of the major antigens in total worm extracts recognized by infection sera (Figs 7–10). The identities of these proteins in tegumental extracts were confirmed by antigen blocking studies (not shown). These results show that Sm29 and SmTsp2 (both monomer and putative dimer) are particularly enriched in the tegumental extracts. In contrast, SmLy6F, and particularly SmLy6B, are less enriched in the tegument extracts. This result is consistent with localization studies showing that SmLy6B is widely distributed throughout schistosomes, including the tegument (SmCD59.2 [20]).

## Discussion

The use of non-reducing SDS-PAGE for the resolution of schistosome antigens substantially changes the nature of western blot data generated on filters probed by the sera of infected mice, rats and humans when compared to standard reducing gels. Studies reported here reveal that a major portion of serum antibodies from *S*. *mansoni* infected mice, rats and humans recognize reduction sensitive epitopes on a limited number of antigens in total extracts of schistosomula and adult worms. Using recombinant schistosome antigens expressed in eukaryotic insect host cells, we unambiguously identified dominant antigens as three Ly6 family proteins- SmLy6B, SmLy6F and Sm29; and two tetraspanins- Sm23 and SmTsp2. Of course it remains possible that other major conformational antigens are present in the schistosome extracts, but not observed on western blots because their epitopes are denatured by SDS-PAGE, perhaps because their conformations are not stabilized by the presence of disulfide linkages. While many other antigens not observed in our studies are known to be recognized by schistosome infection sera, the titers of these antibodies must be relatively low and/or the antigens present in low abundance in total worm extracts such that their signals are low or undetectable under the conditions we used. The parasite may even benefit by somehow biasing the host response away from some antigens, for example nutrient transporters or sensing receptors, against which a robust antibody response could be harmful to parasite survival.

All of the major antigens identified in this study are predicted to be membrane proteins and all except SmLy6F have been reported to be associated with the schistosome tegument in proteomic studies [26, 27]. Notably, SmLy6F has not been identified in any schistosome fractions through proteomics despite its predicted abundance [20], perhaps because it is small, with several predicted N-linked glycosylation sites. Two of these antigens, SmTsp2 and Sm29, are currently strong candidates as vaccine immunogens to prevent schistosomiasis [8, 13, 14] and we previously showed that both of these antigens have epitopes exposed on the surface of living schistosomes [15]. SmLy6B was shown to be a surface tegumental GPI-anchored protein due to its susceptibility to worm surface cleavage by phospholipase C [21]. These observations seem to suggest that host antibody responses during schistosome infection are targeted largely to a limited number of conformational epitopes present on tegumental membrane proteins, particularly those present at the host/parasite interface. The presence of glycosylation on some of these antigens may help limit epitope availability.

Finding two tetraspanins, Sm23 and SmTsp2, as major antigens recognized by *S*. *mansoni* infection serum, was not surprising. Both of these proteins have long been identified as important schistosome antigens [8, 28], and SmTsp2 is a particularly promising vaccine antigen [8]. More surprising was the observation that the prominent epitopes are reduction-sensitive and infection sera recognition of SmTsp2 can be largely blocked with a single scFv (Teg4). These results suggest that tetraspanin recognition by infection sera involves a limited number of conformational epitopes. The functions of Sm23 and SmTsp2 remain unknown, although a role for SmTsp2 in tegumental turnover has been inferred [29].

The identification of three Ly6 family proteins as dominant schistosome antigens was unexpected as, prior to this study, only Sm29 had been identified as an antigen during schistosome infections [9]. The two other Ly6 family proteins identified as major antigens in these studies are SmLy6B and SmLy6F. Expression of both proteins is dramatically upregulated following transformation of cercariae to schistosomula [20, 30]. These proteins are both related to the complement inhibiting protein CD59, though SmLy6B was tested and shown not to possess this activity when expressed on recombinant mammalian cells [20], while SmLy6D remains untested. To date, no evidence as to the functions of these proteins has been experimentally demonstrated despite their immunogenicity during schistosome infection and the identification of at least eight other schistosome Ly6 proteins encoded in the genome [18].

The results of this study, and our previous study [15], clearly show that schistosome infections result in a high titer of antibodies capable of binding to the living schistosomes. In the prior study, we found that infection sera from both mouse and rat clearly stained the surface of living schistosomula and lung worms. The staining was apparent only with infection sera, not pre-infection sera, showing that this staining was not non-specific binding to the antibody Fc domain [31]. In this study, we find high titers of antibodies that recognize known tegumental surface antigens, SmLy6B, SmTsp2 and Sm29, and recognition of SmTsp2 and Sm29 can be largely blocked by pre-treatment with Teg1 and Teg4, scFvs known to recognize living schistosomes [15]. These findings seem to contradict earlier reports that schistosomes bound little host antibody [32, 33], although results would be affected by any denaturants that destroy conformational epitopes. Our findings also seem inconsistent with multiple literature reports showing that humoral responses to tegumental antigens can be protective [5, 6, 33–35]. We find that a very similar host humoral response to the schistosome tegument occurs in both permissive (humans and mice) and non-permissive hosts (rats), suggesting that the presence of these anti-tegument antibodies have little demonstrable impact on the progression of schistosomiasis. We conclude that differences in susceptibility between rats, mice and humans are unlikely due to differences in their humoral responses to major tegumental antigens although differences in isotype production in different species might be important. In addition, we cannot exclude that humoral responses to less abundant surface antigens could be protective, particularly neutralizing antibodies targeting essential parasite functions.

The results reported here have significant implications for schistosomiasis vaccine development. Most obviously, the results imply that induction of an antibody response to the major surface tegument proteins through vaccination may not be protective on its own, because a response of this type is already present following natural infection within permissive hosts such as mice and humans. A similar humoral response to these major conformational antigens was found among human patients that display evidence of both susceptibility and resistance to schistosome infection, suggesting that these antibodies provide no obvious protective benefit to the host. Yet several reports show that two of the major antigens we find to be recognized by serum antibodies following schistosome infection, SmTsp2 and Sm29 [8, 13, 14], are also among the most promising vaccine antigens. One likely explanation is that these vaccines are effective because they elicit a cell-based immune effector response targeting the host-interactive schistosome tegument. These vaccine immunogens are typically produced in prokaryotic hosts which often do not reproduce the conformation of the native proteins and thus the humoral response they elicit is likely poor at binding to live schistosome *in vivo*. These same vaccine immunogens, though, are known to be capable of eliciting a Th1 response that can be harmful to schistosomes [13, 14], and it is likely that this is primarily responsible for the reduction in parasite load following vaccination. It will be interesting to compare the potency of SmTsp2 or Sm29 vaccines prepared in prokaryotic host cells vs conformationally native antigens produced in a eukaryotic host.

The results reported here seem to suggest that efforts to identify schistosome vaccine candidates that promote antibody-mediated damage to worms should focus on antigens that are not among the five antigens we identify as targets of major conformation-dependent antibodies within infection sera. These findings also suggest that antibody binding to the tegument is, in itself, insufficient to result in host immune damage to the parasites and protection from infections. Thus, our findings seem to best support the use of an ‘Achilles Heel’ (also called "Waksman's postulate") approach [36, 37] in which the vaccine target would be a host-exposed functional antigen, essential to worm survival, that is not naturally immunogenic. Antibodies generated to such targets through vaccination should block the function of this antigen, thereby damaging the parasite and protecting the host from future schistosome infections.

A striking finding of these studies is the surprising predominance of antibodies in schistosome infection serum recognizing a limited set of reduction-sensitive epitopes, particularly those on SmTsp2 and Sm29. These epitopes proved to be poorly reproduced when these antigens were expressed by *E*. *coli*. For example, Supplemental S1 Fig shows that the major antigens in schistosome extracts generate strong signals on western blots with rat infection serum despite loadings too low for detection by conventional protein staining. In contrast, a recombinant version of SmLy6-2 produces a barely detectable, reduction-sensitive band on an infection serum blot despite an easily detected protein band. Similar results were obtained with the other SmLy6 proteins, including Sm29, and the recombinant proteins themselves were found to largely self-aggregate (not shown). An exception to this was SmTsp2 in which the *E*. *coli* recombinant protein did produce a strong, reduction-sensitive band on a western blot with infection serum. As another example, we attempted to express Sm29 in insect cells by replacing the schistosome leader sequence with a leader sequence from insect melittin, and even this minor change resulted in a protein that was unrecognized by the Teg4 scFv and much more poorly recognized by infection sera (not shown). These results highlight the importance of using conformationally-native immunogens when preparing antibodies for detection of schistosome tegumental antigens under natural conditions, such as for *in situ* localization or live worm staining.

It is interesting to speculate whether our surprising finding of a strong apparent bias to a limited subset of reduction sensitive tegumental epitopes in the humoral response during schistosome infection has implications with regard to schistosome host immune evasion. The subject of immune evasion by schistosomes has been extensively investigated (reviewed by [38]) and the studies have suggested that schistosomes employ evasion strategies such as minimal protein exposure at the tegument surface, host antigen masking, epitope concealment by carbohydrates, poor immunogenicity of essential antigens, induction of blocking antibodies, secretion of immune modulators and rapid tegument turnover. Our results suggest that schistosomes may somehow elicit a strong antibody response to a limited set of surface antigens on which antibody binding is well-tolerated and perhaps even somehow beneficial to the parasite. For example, these anti-tegumental antibodies could serve as ‘blocking Abs’ [39] that prevent induction of a damaging antibody response. It is worth noting that any agent that induces a minor conformational change to the epitope targeted by these anti-tegumental antibodies will result in the dissociation of the antibody, and schistosomes may somehow exploit this feature to evade immune damage.

## Materials and Methods

### Ethics statement

All studies followed the Guide for the Care and Use of Laboratory Animals of the National Institutes of Health and were approved by the Tufts University Institutional Animal Care and Use Committee (IACUC) under Protocol # G2015-113. This protocol adheres to the National Institutes of Health’s *Public Health Service Policy on Humane Care and Use of Laboratory Animals*. The protocol for the use of human sera was previously approved by the Ethical Committee of the Federal University of Minas Gerais and the patients or their legal guardians provided written informed consent after receiving an explanation of the protocol.

### Reagents

Horseradish peroxidase HRP-conjugated anti-E-tag antibodies were purchased from Bethyl Labs; HRP-conjugated anti-rat IgG, HRP-conjugated anti-mouse IgG, and HRP-conjugated anti-human IgG were purchased from Santa Cruz.

### Schistosome infection sera

The rat infection sera were obtained by performing two *S*. *mansoni* infections of non-permissive Fischer rats, each with 1,000 cercariae, performed 8 weeks apart as previously described [15], and the serum was drawn four weeks after the second infection. Two preparations of mouse infection sera were employed, each obtained from two groups of 10 Swiss-Webster mice infected with 120 cercariae nine weeks earlier. All of the mice survived and contained adult worms at the time the serum was obtained. The pools of serum were prepared by mixing equal amounts of serum from each infected mouse. All human infection sera were obtained from individuals living in two different endemic areas for schistosomiasis (“Melquiades” and “Côrrego do Onça”, Minas Gerais, Brazil). Four different pools of serum (previously reported in [13]) were prepared that each included serum from eight patients. Patients in the pools included sixteen that had egg counts at the time of infection (eight that were recently treated with praziquantel), and sixteen that were stool negative despite known exposure to water contaminated with cercariae (eight there were recently treated with praziquantel). These infected individuals were examined for *S*. *mansoni* infection using the Kato–Katz technique and were negative for other helminthic infections as previously described [13].

### Schistosome extracts

Five day old schistosomula were obtained from the Biomedical Research Institute (Rockville, MD, USA). The schistosomula were produced by in vitro transformation of cercariae and five days of tissue culture following the procedure of [40]. Schistosomula were homogenized in PBS, briefly sonicated to shear the DNA, and diluted in non-reducing SDS buffer. Adult worms were recovered by perfusing infected Swiss Webster mice at 6–7 weeks post infection. Parasites’ extracts were prepared by homogenizing the adult parasites in PBS on ice. Extracts were briefly sonicated to shear the DNA then diluted in non-reducing SDS buffer. Preliminary Western blot experiments were run to determine the appropriate amount of extract to be used in subsequent experiments.

### Production of recombinant proteins in *E*. *coli*

The rat scFv proteins [15, 19] were expressed in *E*. *coli* host cells as fusions with an amino terminal *E*. *coli* thioredoxin to facilitate folding, with a hexa-histidine tag for purification and a carboxyl terminal E-tag epitope for detection. The expression, purification and quantification were performed as previously described for VHH antibody proteins [41].

### Production of recombinant schistosome membrane proteins in insect cells

Synthetic DNA encoding SmLy6A, B, C, F, SmTsp-2, Sm23 and Sm29 was ordered from GenScript Inc in which the codon usage is optimized by the manufacturer for insect expression. Restriction sites were included to facilitate simple ligation into the pFastBac vector (Invitrogen). For SmLy6 proteins, the natural schistosome signal sequence (predicted by SignalP) was replaced with the insect melittin signal sequence (MKFLVNVALVFMVVYISYIYA). Immediately downstream from the signal sequence, we included the following spacer coding sequence (AADYKDDDDKGGGGS) which includes a Not1 cloning site, FLAG epitope and an enterokinase cleavage site. The schistosome mature protein coding sequences followed through the carboxyl terminus. For SmTsp-2, Sm23 and Sm29, the synthetic coding DNA included the complete schistosome protein including leader sequence. We made an additional construction in which the natural signal peptide for Sm29 was replaced with the melittin signal sequence and the spacer sequence described above.

The synthetic coding DNAs were ligated into pFastBac and engineered for insect cell expression using the Bac-to-Bac system (Invitrogen) following the procedures recommended by the manufacturer. Typically, the first virus supernatant was used to infect Sf9 cells at an estimated MOI of 0.1 and cells were harvested 3–5 days post-infection. Recombinant insect cell pellets were either solubilized directly into non-reducing SDS Laemmli buffer (BioRad), or for use in western blot competition studies, we employed a non-reducing insect cell lysis buffer (1% Triton-X-100, 10mM Tris pH 8.0, 140 mM NaCl, 1x Sigma protease inhibitor cocktail).

### Production of recombinant schistosome membrane proteins in *E*. *coli*

DNA encoding the predicted extracellular domains of SmLy6B, C, F, SmTsp-2, and Sm29 was amplified by PCR and ligated into a pET32 E. coli expression plasmid in frame with the *E*. *coli* thioredoxin (Trx) coding DNA and containing hexahistidine and epitope tags. Coding DNA cloning, expression and purification of these recombinant schistosome proteins were all performed by standard methods as previously reported [41].

### Western blotting

Proteins were resolved by SDS-PAGE using AnyKd mini-PROTEAN TGX gels from BioRad under reducing (1.5% βME unless otherwise specified) or non-reducing conditions. Proteins were transferred from gels to Immobilon-P PVDF membranes (Fisher) using a BioRad semi-dry transfer apparatus. NEB Color Prestained Protein Standard, Broad Range (11–245 kDa), which contains only trace reducing agent, was used for estimating molecular weight and to guide the excision of filter strips. Membranes were blocked with 5% non-fat dry milk, 0.1% Tween-20 in PBS (mPBS) for 30 minutes at room temperature or overnight at 4°C. Primary antibody incubations (dilutions indicated in figure legends) were overnight at 4°C in fresh mPBS, and secondary antibody incubations (as recommended by manufacturers) were for one hour at room temperature, all followed by 3 washes, 5–10 minutes each, in PBS, 0.1% Tween-20 (PBST). In some cases, the filters were incubated for 1 hour at room temperature in mPBS 0.1% β-mercaptoethanol. The resulting immunoblots were developed using the ECL Western Blotting detection system from GE Healthcare, as recommended by the manufacturer, and imaged on a BioRad ChemiDoc Touch instrument.

## Supporting Information

S1 Fig Comparison of rat infection serum recognition of schistosome extracts and schistosome antigens expressed in recombinant *E*. *coli*. Total extract of adult mammalian-stage *Schistosoma mansoni* worms, or purified recombinant proteins representing the extracellular domains of either schistosome antigen SmLy6-2 or SmTsp2 expressed by *E*. *coli* host cells (with an *E*. *coli* thioredoxin (Trx) fusion partner), were resolved by SDS-PAGE under non-reducing or reducing conditions. The same loadings of each antigen preparation were run in replicate, then either stained with Coomassie Blue to identify protein species (Protein) or transferred to filters and probed with 1:2000 rat infection sera (Rat sera). Numbers indicate the positions of migration of molecular mass markers (kDa).
(TIF)
