# Supplementary figures and images for: Schistosoma mansoni Infection of Mice, Rats and Humans Elicits a Strong Antibody Response to a Limited Number of Reduction-Sensitive Epitopes on Five Major Tegumental Membrane Proteins
Source: PLoS Negl Trop Dis. 2017 Jan 17;11(1):e0005306. doi: 10.1371/journal.pntd.0005306 (PMC5271416; doi:10.1371/journal.pntd.0005306)

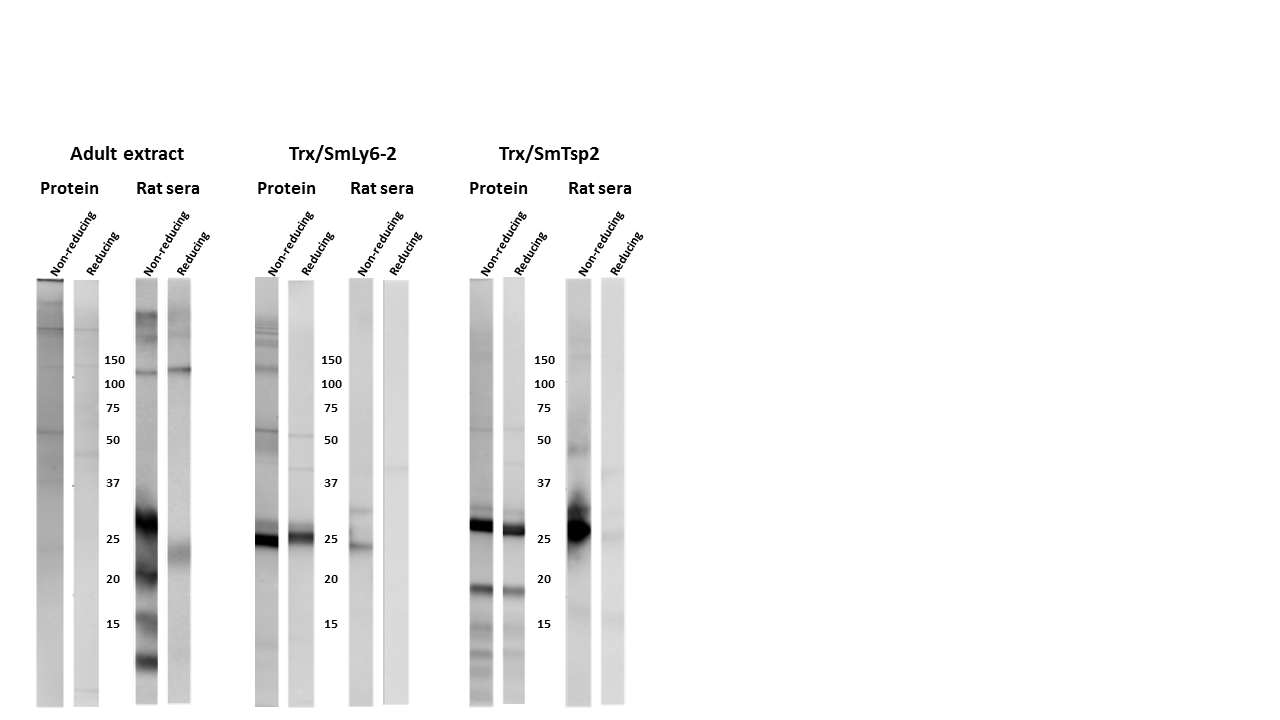

Supplement: S1 Fig — Total extract of adult mammalian-stage Schistosoma mansoni worms, or purified recombinant proteins representing the extracellular domains of either schistosome antigen SmLy6-2 or SmTsp2 expressed by E. coli host cells (with an E. coli thioredoxin (Trx) fusion partner), were resolved by SDS-PAGE under non-reducing or reducing conditions. The same loadings of each antigen preparation were run in replicate, then either stained with Coomassie Blue to identify protein species (Protein) or transferred to filters and probed with 1:2000 rat infection sera (Rat sera). Numbers indicate the positions of migration of molecular mass markers (kDa). (TIF) [file pntd.0005306.s001.tif]
